# Quality of life and acquired organ damage are intimately related to activity limitations in patients with systemic lupus erythematosus

**DOI:** 10.1186/s12891-015-0621-3

**Published:** 2015-08-12

**Authors:** Mathilda Björk, Örjan Dahlström, Jonas Wetterö, Christopher Sjöwall

**Affiliations:** Rehabilitation Center and Department of Clinical and Experimental Medicine, Linköping University, Linköping, Sweden; Department of Rehabilitation, School of Health Sciences, Jönköping University, Jönköping, Sweden; Swedish Institute for Disability Research, Department of Behavioural Sciences and Learning, Linköping University, Linköping, Sweden; Rheumatology/AIR, Department of Clinical and Experimental Medicine, Linköping University, Linköping, Sweden

**Keywords:** Systemic lupus erythematosus, Disease burden, Organ damage, Disability, Quality of life, Activity limitation, Disease phenotype

## Abstract

**Background:**

Systemic lupus erythematosus (SLE) is an autoimmune multi-organ disease, characterized by episodes of disease flares and remissions over time, which may restrain affected patients’ ability to perform daily activities. The purpose of the present study was to characterize variation in activity limitations among well-defined SLE patients, and to describe disease phenotypes, acquired organ damage and their relations to activity limitation and self-reported health, respectively.

**Methods:**

The disease phenotypes were organized into 4 different clinical groups and logistic regression analyses were used to identify how an elevated health assessment questionnaire (HAQ) score was related to disease variables such as phenotypes, disease activity and damage accrual. Correlation and multiple linear regression analyses were used to examine the association between each group of variables – background variables, disease variables and self-reported measurements – and the degree of elevated HAQ.

**Results:**

We found a higher proportion of activity limitation in patients with skin and joint involvement compared to others. The presence of activity limitation, as detected by the HAQ instrument, was significantly associated with quality of life (EuroQol–5D) and accrual of organ damage using the Systemic Lupus International Collaborative Clinics/ACR damage index.

**Conclusions:**

The findings highlight the differing requirements of the multi-professional rehabilitation interventions for the various SLE phenotypes in order to optimize the clinical care of the patients.

## Background

Systemic lupus erythematosus (SLE) is a chronic inflammatory condition characterized by multiple organ involvement, production of antibodies against nuclear constituents and deposition/formation of immune complexes in the affected organs [1]. The clinical spectrum of various disease phenotypes is remarkably diverse and this constitutes a challenge, both in terms of clinical assessment, pharmacological and non-pharmacological treatment. This is furthermore of major concern since periods of uncontrolled disease as well as medical side-effects over time may result in irreversible organ damage [2]. The current treatment strategies with glucocorticoids and disease-modifying anti-rheumatic drugs (DMARDs) intend to relieve symptoms, induce remission, or at least allay the disease activity, prevent future flares and subsequent damage accrual [3].

SLE often affects relatively young patients who are in their most productive years of life [4], and consequences such as disability including work loss [5], activity limitations [6, 7], perceived mental and physical exhaustion [8] and reduced quality of life (QoL) [9–11] are commonly found. Although new treatment options for SLE have recently become available [12], patients continue to report disability. Previous studies report that associations between perceived QoL and disease activity or disease severity are not clear-cut [13–16]. Instead, many authors conclude that the disease burden in SLE is multi-dimensional and has important physical as well as mental aspects [11, 13, 17, 18].

To be able to optimize the rehabilitation efforts for SLE patients, the knowledge about what is related to activity limitations and self-reported health needs to be expanded. Despite that more than 60 % of the patients with SLE have either periodically or permanently reduced ability to perform daily activities [19], it has to our knowledge not been studied whether activity limitations and disease manifestations are related or not. An early study by Milligan *et al.* [20] displayed that activity limitations as measured by the health assessment questionnaire (HAQ) were not related to disease activity in female SLE patients, which could possibly be confounded by HAQ being more related to other aspects of disability [6], and/or confounders in self-reporting. A reduced health-related quality of life (HRQL) assessment in SLE patients, on the other hand, was shown to be associated with musculoskeletal impairments [21]. These findings imply that disability and self-reported health in SLE may be better explained by measures other than global disease activity, *e.g.,* acquired organ damage, number of involved organ systems or disease phenotype.

Thus, the aims herein were to characterize variation in activity limitations among well-defined SLE patients, and to describe disease phenotypes, acquired organ damage and their relations to activity limitation and self-reported health, respectively.

## Methods

### Patients & laboratory analyses

In total, 192 SLE patients included in a prospective project with structured follow-up at the Rheumatology clinic, Linköping university hospital, Sweden, were included in the present cross-sectional study. The study was based on data from the most recent visit to the rheumatologist during 2011. The patients were recruited consecutively without regard taken to present disease activity. Most were prevalent cases (93 %), but some (7 %) had newly diagnosed SLE at the time-point of data collection. One hundred and fifty four patients (80 %) fulfilled the 1982 ACR classification criteria [22], whereas 38 (20 %) had a clinical diagnosis of SLE based on a history of abnormal antinuclear antibody (ANA) titre by immunofluorescence microscopy *plus* at least two typical organ manifestations (referred to as the Fries’ diagnostic principle) [23]. One hundred and seventy patients (89 %) fulfilled the 2012 Systemic Lupus International Collaborative Clinics classification criteria [24]. The study population has recently been described in detail [25].

Laboratory analyses included erythrocyte, leukocyte and platelet counts, urinalysis, erythrocyte sedimentation rate (ESR), high sensitivity C-reactive protein (CRP), creatine kinase, creatinine and plasma complement proteins (C3, C4). IgG class antibodies with reactivity against double-stranded (ds) DNA were detected by the *Crithidia luciliae* microscopy test; 56/192 (29 %) individuals were positive at the time-point of data collection, whereas altogether 44 % had been anti-dsDNA antibody positive at least once (cut-off titre of 1:10, corresponding to >99^th^ percentile among healthy female blood donors was used) [26].

### Primary outcome measure

The validated Swedish version of HAQ [27] measuring self-reported activity limitation was the primary outcome. The HAQ consists of 20 questions representing common daily activities. The response alternatives for each of the 20 questions were ‘without any difficulty’ (score = 0), ‘with some difficulty’ (score = 1), ‘with much difficulty’ or ‘with use of an assistive device’ (score = 2), and ‘unable to do’ (score = 3). The highest score obtained for any question of a given subcategory determines the score for the subcategory. A total score (0–3) was calculated based on the sum of the scores for the various subcategories divided by the number of subcategories that were answered. The Swedish version of HAQ is well-established psychometrically tested with good results [28] and widely used also in SLE [6, 29].

### Background variables

Background variables concerning age, sex and disease duration were collected. The use of anti-rheumatic drugs, including antimalarials, other DMARDs and glucocorticoids was registered.

### Disease variables

The number of fulfilled American Collegue of Rheumatology (ACR) criteria was registered and the study population was organized into different disease phenotypes based on the 1982 ACR classification criteria (*i.e.,* skin disease, arthritis, renal or hematologic disorders) [22]. Acquired organ damage was estimated using the validated Systemic Lupus International Collaborative Clinics/ACR damage index (SDI), which covers 12 organ systems and measures accumulated organ damage that has occurred since the onset of SLE. SDI is scored regardless of whether the damage can be attributed to SLE or to other causes [2, 30]. Disease activity was recorded by the SLE disease activity index 2000 (SLEDAI-2 K), both with and without index modification by the exclusion of laboratory items for hypocomplementemia and anti-dsDNA antibody binding [31]. In addition, the physician’s global assessment (PGA; scored 0 = remission and to 4 = maximum disease activity) of perceived disease activity was registered [32].

### Self-reported measures

Four patient self-reported measures were used to capture a wide range of disability and health; pain intensity, activity limitation, QoL and well-being. Pain intensity, defined as ‘the experienced pain because of your SLE during the last week’, was self-reported on a 0–100 mm visual analogue scale (VAS) ranging from 0 (no pain at all) to 100 (worst possible pain). Wellbeing was estimated in the same manner, with 0 representing ‘best possible wellbeing’ and 100 ‘worst possible wellbeing’ [33]. Generic HRQL was measured using EuroQol–5D (EQ5D) that provides a profile of the self-reported problems based on five different dimensions [34]. EQ5D is useful and reliable to predict HRQL in different conditions [35].

### Statistical analysis

Disease phenotypes were organized into 4 different clinical groups; skin disease (ACR criteria No. 1–3), arthritis (ACR criterion No. 5), renal disorder (ACR criterion No. 7) and hematologic disorder (ACR criterion No. 9) [22]. Differences in proportions between patients with activity limitation (HAQ > 0) and patients without activity limitation (HAQ = 0) were examined by Chi-square tests of proportions and differences in levels between the two groups were examined by Mann–Whitney *U* tests.

Associations between the variables and HAQ were examined in a two-step procedure: (1) examination of variables associated with elevated HAQ score (1 = elevated HAQ, 0 = no elevated HAQ), and (2) examination of variables associated with degree of elevated HAQ given that patients have elevated HAQ.

Step 1: Correlations were examined between elevated HAQ and each group of variables (background variables, disease variables and self-reported measures). To further examine the overall association between elevated HAQ and all groups of variables (background variables, disease variables and self-reported measures) the variables that correlated significantly with elevated HAQ were used in multiple logistic regression analyses, first for each group of variables and thereafter for all variables (using a stepwise forward procedure where at each step the variable which added most to the model was added until no more variable could be added with *p* < 0.05).

Step 2: Given an elevated HAQ, correlations were then examined between degree of activity limitation and each group of variables (background variables, disease variables and self-reported measures). To further explore combined correlations (*i.e.,* shared variance) between the variables and degree of elevated HAQ, variables that correlated significantly with elevated HAQ were put into multiple linear regression analyses (using a stepwise forward procedure where at each step the variable which added most to the model was added until no more variable could be added with *p* < 0.05).

All analyses were done using IBM SPSS version 20.0. *P*-values < 0.05 were considered significant.

### Ethics and consent

Oral and written informed consent was obtained from all subjects and the patient anonymity has been preserved. The study protocol was approved by the regional ethical review board in Linköping (Decision No. M75–08/2008).

## Results

**Fig. 1:**
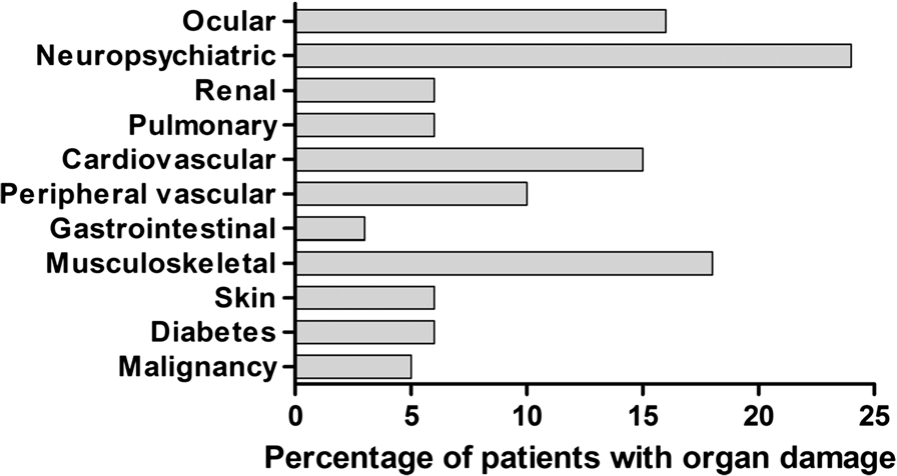
Distribution of organ damage. The figure illustrates the distribution of damage accrual for each domain according to the Systemic Lupus International Collaborative Clinics/ACR damage index. Of the 192 included patients, 106 (55 %) showed acquired organ damage in at least one organ domain

The study population consisted of 192 patients, whereof 172 were women (mean age, 52.3 years; range, 18–87) and 20 were men (mean age, 56.0 years; range, 27–90). Data on background variables, disease variables and self-reported measures for all patients are given in Table 1. The distribution of acquired organ damage (SDI) in the study population is shown in Figure 1.

**Table 1:** Patient characteristics. Characteristics of the patients in relation to the presence of activity limitations

*Characteristics*	*Mean (standard deviation) [range], or %*			p-value***
	Total (*n* = 192)	HAQ > 0 (*n* = 117)	HAQ = 0 (*n* = 75)	
*Background variables*				
Age (years)	52.7 (17.4) [18–90]	56.8 (17.1) [18–90]	46.2 (16.0) [21–87]	<0.0001
Females	89.6 %	90.6 %	88.0 %	n.s.
Caucasian ethnicity	92.5 %	92.9	92.0	n.s.
Disease duration (years)	13.4 (10.2) [0–48]	14.3 (11.3) [0–48]	12.0 (8.2) [0–38]	n.s.
*Continuous medication*				
AM as only DMARD	40.6 %	41.0 %	40.0 %	n.s.
Other DMARD ± AM	29.7 %	30.8 %	28.0 %	n.s.
Glucocorticoids	58.9 %	70.9 %	40.0 %	<0.0001
*Disease variables*				
*Fulfilled ACR criteria* (*n*)	4.6 (0.3) [3–9]	4.5 (1.2) [3–8]	4.7 (1.4) [3–9]	n.s.
1. Malar rash	43.2 %	47.0 %	37.3 %	n.s.
2. Discoid rash	16.1 %	20.5 %	9.3 %	0.04
3. Photosensitivity	52.6 %	54.7 %	49.3 %	n.s.
4. Oral ulcers	9.4 %	9.4 %	9.3 %	n.s.
5. Arthritis	76.6 %	76.9 %	76.0 %	n.s.
6. Serositis	39.1 %	36.8 %	42.7 %	n.s.
7. Renal disorder	20.8 %	17.1 %	26.7 %	n.s.
8. Neurologic disorder	4.2 %	4.3 %	4.0 %	n.s.
9. Hematologic disorder	51.6 %	42.7 %	65.3 %	0.002
10. Immunologic disorder	46.9 %	44.4 %	50.1 %	n.s.
11. Antinuclear antibody*	98.4 %	97.4 %	100 %	n.s.
SDI	1.4 (1.9) [0–8]	1.9 (2.1) [0–7]	0.79 (1.39) [0–8]	0.0001
SLEDAI-2 K	2.3 (3.3) [0–24]	2.3 (3.7) [0–24]	2.3 (2.7) [0–11]	n.s.
Modified SLEDAI**	1.0 (2.5) [0–20]	1.2 (2.9) [0–20]	0.81 (1.96) [0–10]	n.s.
Physician’s global assessment (PGA)	0.26 (0.55) [0–3]	0.29 (0.62) [0–3]	0.20 (0.43) [0–2]	n.s.
*Self-reported measures*				
Pain intensity (mm)	29.0 (27.1) [0–100]	38.5 (27.0) [0–100]	13.0 (18.5) [0–73.2]	<0.0001
HAQ (0–3)****	0.44 (0.60) [0–3.0]	0.73 (0.62) [0.13–3.0]	0	
EQ5D	0.67 (0.30) [−0.35–1]	0.56 (0.31) [−0.35–1]	0.84 (0.18) [0–1]	<0.0001
Well-being (mm)	29.8 (25.8) [0–100]	39.2 (24.7) [0–100]	13.6 (18.8) [0–74.2]	<0.0001

*Abnormal titre of ANA by immunofluorescence microscopy

**SLEDAI–2 K indicated by the exclusion of laboratory items for hypocomplementemia and anti-dsDNA antibody binding

***Performed with Mann–Whitney *U* test or Chi-square test (where appropriate)

****Primary outcome measure

AM = Antimalarials; DMARD = disease modifying anti-rheumatic drugs; SDI = Systemic Lupus International Collaborative Clinics/ACR damage index; SLEDAI = SLE disease activity index–2 K; HAQ = Health Assessment Questionnaire

EQ5D = EuroQol–5D; n.s. = not significant

**Fig. 2:**
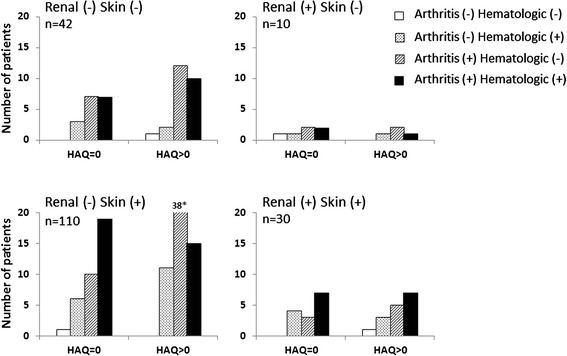
Disease phenotype *vs.* activity limitation. Distribution of patients reporting activity limitation (HAQ) in the different clinical groups. Patients were divided with regard to disease phenotypes (ACR-82 criteria No. 1–3 = skin disease; No. 5 = arthritis; No. 7 = renal disorder; and No. 9 = hematologic disorder). The graph should be read as, for example, in the lower right panel patients with renal and skin disease are further categorized into arthritis and/or hematologic manifestations. * actual value for HAQ > 0 is 38

The group that reported any difficulty in performing daily activities (HAQ > 0) consisted of 117 patients (61 %). This group was significantly older, used glucocorticoids to a higher extent, had more damage accrual and frequently fulfilled the ACR classification criteria for discoid rash. However, the group with elevated HAQ had less of the hematologic phenotype compared to the group where the HAQ score was zero (Table 1). In addition, the group with difficulties in performing daily activities reported significantly more pain, but lower QoL and well-being (Table 1). The distribution of patients with different phenotypes and/or elevated *vs.* no elevated HAQ scores is shown in Fig. 2; observe the high frequency of patients (n = 38) characterized by skin/joint involvement, without renal/hematologic phenotype, and elevated HAQ scores. Patients meeting ACR criteria for hematologic disorder had less of the arthritic phenotype (*p* =0 .003, Cramer’s V = 0.21), and patients without difficulties in performing daily activities were more often associated with the hematologic phenotype (*p* = 0.002, Cramer’s V = 0.22).

Using the results from distributions of cases according to phenotype and elevated HAQ scores, only the absence of hematologic phenotype was found to be significantly related to elevated HAQ in the logistic regression (Table 2). In background variables, age was significantly associated with elevated HAQ score, and among the disease variables this was also the case for SDI. In the self-reported measures, both EQ5D and well-being were significantly correlated with elevated HAQ. Finally, variables that correlated significantly with elevated HAQ, were merged into a final logistic regression analysis (Table 2) showing a Hosmer & Lemeshow R^2^ as well as a Cox & Snell R^2^ of 0.41.

**Table 2:** Activity limitation predictors. Multiple logistic regression models of the effect of clinical measures on the risk of elevated HAQ score among individuals with SLE

			95 % CI for Odds Ratio			R^2^		Predictive ability	
	B (SE)	*P*	LL	OR	UL	H&L	C&S	Sens.	Spec.
Phenotypes						0.04	0.05	1	0
Constant	0.947 (0.231)								
Hematologic	−0.926 (0.306)	0.002	0.217	0.396	0.722				
Background variables						0.07	0.09	0.79	0.45
Constant	−1.49								
Age	0.037 (0.009)	<0.001	1.019	1.038	1.057				
Disease variables						0.06	0.08	0.66	0.60
Constant	−0.006 (0.191)								
SDI	0.375 (0.108)	<0.001	1.178	1.455	1.798				
Self-reported measures						0.26	0.29	0.84	0.76
Constant	1.891 (1.318)								
EQ5D	−3.276 (1.502)	0.029	0.002	0.038	0.717				
Well-being	0.043 (0.014)	0.001	1.017	1.044	1.072				
Overall						0.41	0.41	0.87	0.69
Constant	0.817 (1.434)								
Age	0.031 (0.016)	0.050	0.941	0.970	1.000				
SDI	0.432 (0.194)	.0026	1.053	1.541	2.256				
EQ5D	−3.350 (1.502)	0.026	0.002	0.035	0.666				
Well-being	0.048 (0.014)	<0.001	1.020	1.049	1.078				
Hematologic	−1.709 (0.532)	0.001	0.064	0.181	0.513				

LL = lower limit; UL = upper limit; H&L = Hosmer & Lemeshow; C&S = Cox & Snell; Sens. = sensitivity; Spec. = specificity; SDI = Systemic Lupus International Collaborative Clinics/ACR damage index; EQ5D = EuroQol–5D

Among the patients with activity limitation, a multiple linear regression with the variables that were significantly correlated with HAQ explained 50 % of the variance in HAQ scores. As demonstrated in Table 3, given an elevation of HAQ, EQ5D was identified to have the strongest relation to HAQ, followed by SDI. Even though age, pain and well-being significantly correlated with degree of elevated HAQ, these variables could not add any significant explanation of variance in HAQ elevation than to what could be explained by EQ5D and SDI. Repeated analyses excluding patients with lowest HAQ elevation (to control for somewhat positively skewed data) yielded similar results.

**Table 3:** Predictors of HAQ. Factors predicting impaired high scores of HAQ among patients with elevated HAQ (Multiple Linear Regression Analysis); *n* = 117)

	*B*	*SE B*	*BETA*	*P*	*R*
Step 1					
Constant	1.444	0.091			
EQ5D	−1.285	0.143	−0.642	<0.001	−0.64
Step 2					
Constant	1.132	0.106			
SDI	0.099	0.021	0.334	<0.001	0.52
EQ5D	−1.054	0.140	−0.527	<0.001	−0.64
Excluded variables					
Age					0.29
Well-being					0.41
Pain					0.36

*Note*: *R* ^2^ = 0.41 for Step 1, Δ*R* ^2^ = 0.10 for Step 2 (*p* < 0.001)

*B* is the regression coefficient, *SE B* the standard error of *B*, *BETA* is the standardized regression coefficient

*r* = Pearson correlation between independent variables and HAQ scores

## Discussion

Activity limitations in the daily life constitute major problems for individuals with SLE [13]. This cross-sectional cohort study of 192 well-characterized patients provides evidence for a higher proportion of activity limitation in SLE patients with skin and joint involvement compared to others. In addition, we found that the presence of activity limitation as detected by the HAQ instrument was significantly related to QoL (EQ5D) and acquired organ damage (SDI). Since previous research has, to our knowledge, not been focusing on the relation between organ damage and self-reported aspects in well-defined and established SLE our findings are novel. SLE patients have reported different types of discomfort related to involvement of organs and also their body to be unpredictable in how their daily activities and health are affected by the disease [36]. Our findings add knowledge to the relation between the different phenotypes of the disease and the experience of SLE in daily life. This underlines the different requirements of the multi-professional rehabilitation interventions for the various SLE phenotypes in order to optimize the clinical care of the patients.

Although the HAQ instrument was originally developed for rheumatoid arthritis (RA) [37], it has also been employed for SLE [6, 16, 17, 20, 29], and represents a useful measure of activity limitation in disparate rheumatic conditions [27, 38–40]. In our study population the overall mean of HAQ was 0.44, this is in line with previous results on disability in SLE [6, 29] and significantly altered in relation to HAQ in the Swedish general population (HAQ score of 0.08) [41]. In the study by Malcus Johnsson *et al.* [6], 42 % of the patients reported interference with performance of daily activities (HAQ > 0) whereas we found a slightly higher proportion of patients with activity limitations (61 %). The sample in the present study was obviously older (but had similar disease duration), and this could possibly explain some of the difference. Our observation of a distinct relative increase of raised HAQ scores in individuals with skin and joint involvement is not surprising, since this lupus variant may resemble RA in many ways. Gilboe *et al.* used the modified HAQ instrument (MHAQ) [42] and found that activity limitations in Norwegian SLE patients were stable over time and did not predict future organ damage as was assessed by the SDI [17, 43].

Difficulties in performing daily activities have, to our knowledge, not been reported in SLE patients with mainly skin manifestations. However, Goreshi *et al.* [44] reported that 62 % of patients with dermatomyositis had an elevated HAQ score which was also associated with reduced QoL. Thus, based on results from these authors as well as from the present study, patients with skin disease may be underserved in terms of being evaluated by clinicians for activity limitations and self-reported health. In order to evaluate the need for rehabilitation interventions by for example by occupational therapist and physical therapists, HAQ could be used as a part of the clinical routine for patients with skin manifestations.

In the present study, organ damage (SDI), age, QoL (EQ5D) and well-being were significantly correlated with elevation of HAQ score. Among the disease phenotypes, the absence of hematologic disorder had the strongest association with a raised HAQ score. Given that the HAQ instrument is known to cover different aspects of arthritis, and that the hematologic and arthritic phenotypes were inversely associated herein, it could be speculated that it is the presence of arthritis *per se*, rather than the absence of hematologic disorder, which promotes this significance. The HAQ instrument has previously been shown to be strongly associated with well-being and QoL in SLE [6, 38], as well as in other rheumatic diseases [41], which is probably a sign of self-reported measures being closely related in rheumatic diseases [45]. This is seen in our results by EQ5D being highly associated with risk of elevated HAQ. In RA, however, HAQ is only weakly related to disease variables, such as the DAS28 score [41]. In line with the observation of Milligan *et al.*, [20] we found no association between disease activity and activity limitations. In fact, SDI was the only disease variable that significantly explained variance in degree of HAQ elevation (given elevated HAQ). Whereas it is rather easy to capture subtle signs of irreversible organ damage leading to activity limitations in RA by longitudinal radiographic examinations [46], the challenge is greater in SLE. SDI measures the accumulated organ damage in several organ systems that has occurred since the onset of SLE and has been present for at least 6 months regardless of its cause (*i.e.,* caused by disease flares, therapeutic side-effects or concomitant diseases) [30]. Several studies have shown a convincing correlation between SDI and disease outcome, particularly if damage occurs early [47–49]. Thus, SDI covers a broad spectrum of symptoms and sequelae that potentially can have major impacts on the ability to perform daily activities; therefore it is not unexpected that SDI is closely associated with HAQ. In addition, increased damage accrual was recently identified to be an important predictor of the total cost for SLE care in Sweden [50]. However, this study did not have the statistical power to consider which types of damage that were specifically associated with an elevated HAQ score. The individual SDI items have a wide range of variability, some of which are expected to affect HAQ (*e.g.,* deforming arthritis, osteoporosis, osteonecrosis), whereas others are not expected to affect HAQ in the majority (*e.g.,* renal, diabetes and premature gonadal failure).

Related to the finding of age as being significantly related to elevated HAQ score in SLE patients, Poole *et al.* [51] recently found activity limitation to be pronounced in younger parenting females with SLE. Mothers with small children (<5 years) reported that having energy to talk/listen to a child was the most difficult parenting task. Mothers with children older than 5 years of age reported difficulties in playing games, shopping, and doing household chores. A limitation with the HAQ instrument is that it includes only predefined activities focusing on self-care and basic needs. More recently, however, the need for incorporation of the patient perspective in assessment and interventions has been stressed [52] and assessments of a broader range of functioning, including measures of participation has been used [53, 54]. As an extension of the present study these measures could be used to for example capture the activity limitations and preferences expressed by younger women with SLE.

The large Swedish study population with well-organized data and very few internal missing values constitute the strengths of this study. However, although several important conclusions were drawn it also has limitations. In the comparison of patients with an elevated HAQ score and the patients with an unaffected HAQ, the patients with elevation in HAQ had a higher extent of organ damage, were prescribed higher doses of glucocorticoids and were older. Age has earlier been reported to have an impact on HAQ [55], and thus it cannot be excluded that age rather than SLE *per se* account for some of the differences in activity limitation between the two groups. Even though HAQ is not psychometrically tested in SLE it is well established and used in earlier research and also recommended as an outcome measure in SLE [56]. The study with a cross-sectional design, within a more comprehensive prospective project, did not evaluate changes in a longitudinal perspective. To fully explore the relationship between background variables, disease variables and self-reported measures, we warrant future studies monitoring the changes in self-reported measures as disease variables changes over time. Also fatigue and depressive symptoms, as self-reported aspects often altered by SLE should be included to cover a wider range of disability.

## Conclusions

In conclusion, EQ5D and the SDI were shown to have the strongest associations with activity limitations in this Swedish SLE population. These instruments record two completely different aspects of the disease, and this clearly illustrates the complexity of activity limitations in SLE.

## Abbreviations

ACR: American Collegue of Rheumatology
ANA: Antinuclear antibody
CRP: C-reactive protein
DMARDs: Disease-modifying anti-rheumatic drugs
ESR: Erythrocyte sedimentation rate
EQ5D: EuroQol–5D
HAQ: Health assessment questionnaire
HRQL: Health-related quality of life
PGA: Physician’s global assessment
RA: Rheumatoid arthritis
SDI: Systemic Lupus International Collaborative Clinics/ACR damage index
SLE: Systemic lupus erythematosus
SLEDAI-2K: Systemic lupus erythematosus disease activity index 2000
QoL: Quality of life
VAS: visual analogue scale
